# Molecular epidemiology and socio-demographic risk factors of sexually transmitted infections among women in Lebanon

**DOI:** 10.1186/s12879-020-05066-8

**Published:** 2020-05-27

**Authors:** Jessica Hanna, Ruba Yassine, Rana El-Bikai, Martin D. Curran, Mathilde Azar, Joumana Yeretzian, Rana Skaf, Claude Afif, Toufic Saber, Saadeddine Itani, Manal Hubeish, Tamima El Jisr, Fadia Hamzeh, Mira El Chaar

**Affiliations:** 1 Faculty of Health Sciences, University of Balamand, P.O.Box 166378 Ashrafieh, Beirut, 1100-2807 Lebanon; 2 Public Health England Clinical Microbiology Laboratory, Addenbrooke’s Hospital, Cambridge, UK; 3 Faculty of Medicine, University of Balamand, Beirut, Lebanon; 4 Department of Obstetrics and Gynecology, Saint George Hospital University Medical Center, Beirut, Lebanon; 5 Makassed General Hospital, Beirut, Lebanon; 6 National Institution of Social Care and Vocational Training, Beirut, Lebanon

**Keywords:** STIs, HPV genotyping, Surveillance, Molecular detection, Clinical symptoms, Risk factors

## Abstract

**Background:**

Sexually transmitted infections (STIs) cause a major public health problem that affect both men and women in developing and developed countries. The aim of the study was to estimate the prevalence of 11 STIs among women who voluntarily participated in the study, while seeking gynecological checkup. The existence of an association between the presence of pathogens and symptoms and various sociodemographic risk factors was assessed.

**Methods:**

A total of 505 vaginal and cervical specimens were collected from women above 18 years of age, with or without symptoms related to gynecological infections. Nucleic acid was extracted and samples were tested by real-time PCR for the following pathogens: C*hlamydia trachomatis*, *Neisseria gonorrhoeae, Mycoplasma genitalium*, *Ureaplasma urealyticum*, *Urealplasma parvum*, *Trichomonas vaginalis*, *Mycoplasma hominis*, *Mycoplasma girerdii*, *Gardnerella vaginalis*, *Candida albicans and* Human Papillomavirus *(*HPV). Positive HPV samples underwent genotyping using a microarray system.

**Results:**

Of the 505 samples, 312 (62%) were screened positive for at least one pathogen. Of these, 36% were positive for *Gardnerella vaginalis,* 35% for *Ureaplasma parvum*, 8% for *Candida albicans*, 6.7% for HPV, 4.6% *for Ureaplasma urealyticum*, 3.6% for *Mycoplasma hominis,* 2% for *Trichomonas vaginalis*, 0.8% for *Chlamydia trachomatis*, 0.4% for *Mycoplasma girerdii*, 0.2% for *Mycoplasma genitalium* and 0.2% for *Neisseria gonorrhoeae*. Lack of symptoms was reported in 187 women (37%), among whom 61% were infected. Thirty-four samples were HPV positive, with 17 high risk HPV genotypes (HR-HPV); the highest rates being recorded for types 16 (38%), 18 (21%) and 51 (18%). Out of the 34 HPV positives, 29 participants had HR-HPV. Association with various risk factors were reported.

**Conclusions:**

This is the first study that presents data about the presence of STIs among women in Lebanon and the MENA region by simultaneous detection of 11 pathogens. In the absence of systematic STI surveillance in Lebanon, concurrent screening for HPV and PAP smear is warranted.

## Background

Sexually transmitted infections (STIs) cause a major public health problem; they affect both men and women in developing and developed countries. There are more than 30 types of STIs caused by different pathogens including bacteria, viruses and parasites that may differ in their clinical manifestations [1]. STIs are frequently asymptomatic and can lead to various complications such as pelvic inflammatory disease, infertility, ectopic pregnancy, cervical cancer and congenital infections in infants born to infected mothers [2–6]. Estimating the prevalence of STIs is essential to prevent, control and properly treat individuals carrying the infections.

The development of new technologies that include nucleic acid amplification platforms, such as multiplex PCR assays, have improved the diagnosis and reporting of various infections [7, 8]. The burden of STIs has long been underestimated by the public health sector overlooking the “hidden epidemics” of such diseases. However, recently the alarm has been triggered to battle these infections where CDC, WHO and other organizations have conducted global plans which will contribute to a radical decline in new STIs and in deaths related to such infections [9].

There are limited STI surveillance reports published in the MENA region. In Lebanon, routine screening for all potential STIs is cost-prohibitive, limiting the knowledge about transient carriers and transfer of pathogens. Premarital sexual intercourse is becoming more common in Lebanon [10, 11]. This fact is coupled with sexual behavior shifts such as an increase in multiple partners and homosexuality practices, both of which are linked to increased risks of STIs [10–12]. Generally, sexual and reproductive health quality services, such as screening, diagnosis and preventive checkups, are not easily accessible and are cost prohibitive. Women may usually seek medical attention for cycle variations, abnormal vaginal secretions or cervical screening.

The aim of the study was to estimate the prevalence of eleven STIs among women seeking gynecological checkups, evaluate the occurrence of concomitant STIs, and assess the existence of an association between the presence of pathogens and symptoms and various socio-demographic risk factors. Determining the presence of STIs is essential to improve the reporting system in the country and to develop appropriate health care strategies that focus on preventive and protective measures.

## Methods

### Study design

A cross-sectional study was conducted in Beirut, Lebanon between November 2016 and December 2017. An estimated number of 377 subjects was determined using the Raosoft online sample calculator with alpha 0.05 and a confidence interval of 95% [13]. Participants were collected using a convenience sample of 505 women seeking gynecological checkups in private clinics located in three large tertiary hospital centers and two dispensaries in Beirut, Lebanon. These sites receive a high number of patients from various socio-demographic and socioeconomic status.

Women aged 18 years and above, presenting with or without symptoms of possible reproductive tract infections were included. Whereas, women who underwent hysterectomy or claimed not to have any kind of previous sexual intercourse were excluded. Since specimen collection may include a mild invasive procedure, pregnant women were excluded from this study for safety reasons. On the other hand, all nonpregnant women who were approached, voluntarily participated in the study, knowing that the screening was free of charge.

### Ethical consideration

This study was approved by the institutional review board of Saint George Hospital and Makassed General Hospital. A written informed consent was obtained from all eligible women before entering the study. Women were informed about the study, their participation was voluntary and they had the right to withdraw at any moment. The risks and benefits were clearly explained. Results were communicated to the gynecologists who reported them to patients.

### Data collection

The healthcare practitioners, including physicians, midwives or nurses, were responsible for collecting the samples and filling the questionnaires with the patients. Data collection included information about the socio-demographic profile of patients, their lifestyle, habits as well as their medical and sexual history. The questionnaires were completed having less than 10% missing data on some questions except for the item on job occupancy which had 15% missing data. Clinical symptoms such as painful urination, abdominal pain, discharge, itching, painful intercourse, bleeding in urine and bleeding post sexual intercourse were recorded. Clinical symptoms were not filled in 12% (*N* = 61) of the questionnaires. For women who had a positive HPV results in our selected sample, follow-up with their healthcare providers showed that only 17 of them underwent a Papanicolaou (PAP) smear test simultaneously. Data were reported in our results to determine if there are discrepancies between presence of the HPV infection and PAP smear pathological results.

### Sample collection and DNA extraction

Two genital flocking swabs (endocervical and vaginal) were collected from each woman and placed in a single tube containing universal transport media (Copan Diagnostics Inc., Murietta, USA) and stored prior to testing at − 80 °C.

DNA from 200 μl of each sample was extracted from the collected tubes using a QIAamp DNA extraction kit (Qiagen, Hilden, Germany) according to the manufacturer’s protocol. An artificial single stranded molecule was used as an internal control [14] for all samples prior to extraction. DNA was concentrated in 60 μl of elution buffer.

### Multiplex PCR for the detection of ten pathogens

Four multiplex PCR assays that include two quadriplex and two duplex assays were used to test for the presence of 10 pathogens: *Chlamydia trachomatis*, *Neisseria gonorrhoeae*, *Mycoplasma genitalium*, *Ureaplasma urealyticum*, *Ureaplasma parvum*, *Trichomonas vaginalis*, *Mycoplasma hominis*, *Mycoplasma girerdii*, *Gardnerella vaginalis* and *Candida albicans*.

Primers-probes sets were purchased from Metabion (Planegg/steinkirchen, Germany) and Thermofisher (Massachusetts, USA) (Table S1). Positive controls were kindly donated by Addenbrooke’s hospital clinical microbiology laboratory, Cambridge, UK. The 12.5 μl duplex or quadriplex PCR reactions included 12.5 μl of Platinum Quantitative PCR SuperMix-UDG (Invitrogen Carlsbad,CA), 3 mM MgSO4, 6.25 pmol of forward and reverse primers, 2.5 pmol of fluorogenic probes, and 5 μl of purified nucleic acid. The reaction was initiated by two heating steps at 50 °C for 2 min and 95 °C for 2 min followed by 45 amplification cycles with the following conditions: 95 °C for 15 s and 60 °C for 60 s. The appropriate fluorescence reading was performed at 60 °C.

### Detection of HPV genotypes

A real time PCR monoplex reaction that target the L1 open reading frame of HPV was performed using the GP5+/6+ consensus primers pairs (GP5+ primer: 5′-TTTGTTACTGTGGTAGATACTAC-3′ and GP6+ primer 5′-GAAAAATAAACTGTAAATCATATTC-3′) [15]. A universal taqman MGB probe was designed and used for this study: 5′- FAM-CATGNNGARGAATATG-MGB-3′. The PCR reaction was done using Platinum Quantitative PCR SuperMix-UDG as previously described.

Positive HPV samples were screened for HPV genotypes using EUROArray HPV kit (Euroimmun, New Jersey, USA). The kit includes amplification and hybridization steps that identify 30 different HR and LR HPV types. The kit is based on the amplification of E6 and E7 viral oncogenes fragments and their detection via hybridization with immobilized DNA probes [16, 17].

The assay was performed according to the manufacturer’s instructions. The first reaction was a Pre-PCR step that included 10 μl of PCR Mix A and 10 μl of PCR Mix B and 5 μl of the extracted sample DNA. The PCR reactions were performed on the CFX96 real-time PCR machine (Bio-Rad, California, USA). Each PCR product was mixed with 65 μl of Hybridization buffer and incubated at 45 °C for 60 min on a microarray slide. Slides were washed with buffer, dried and read on the Microarray Scanner and EUROArray Scan software that detects signal strength in terms of immunofluorescence units.

### Statistical analysis

Data analysis was performed using SPSS (version 20.0). Descriptive analysis including frequencies, percentages and averages were used when appropriate to describe the population under study. Prevalence was calculated to indicate the number of subjects carrying STIs among the recruited sample. A chi-square test was used to assess the association between exposures and outcomes expressed as categorical variables. The threshold for significance was set at alpha 0.05. Univariate and multivariate logistic regression were used to calculate odds of infection relative to variables that showed significance at the bivariate level. Odds ratios were considered significant if the 95% confidence interval excluded 1.

## Results

### Prevalence of pathogens

Eleven pathogens were identified as causing STI among 62% (*N* = 312) of the participants. Bacteria were identified in 58% (*N* = 295) of the patients; 8.1% were positive for *C.albicans* (*N* = 41), 6.7% for HPV (*N* = 34) and 2% for *T.vaginalis* (*N* = 10). The pathogens causing bacterial infections were 36% for *G.vaginalis* (*N* = 184), 35% for *U.parvum* (*N* = 175), 4.6% for *U.urealyticum* (*N* = 23), 3.6% for *M.hominis* (*N* = 19), 0.8% for *C.trachomatis* (*N* = 4), 0.4% for *M.girerdii* (*N* = 2), 0.2% for *M.genitalium* (*N* = 1), and 0.2% for *N.gonorrhoeae* (*N* = 1) (Table 1).

**Table 1:** Total number of STIs detected in 505 women

**A**											
	**Prevalence of STI’s pathogens**										
**Pathogens**	**CT**	**NG**	**MG**	**MGI**	**MH**	**UU**	**UP**	**GV**	**CA**	**TV**	**HPV**
**CT**	**4**	1	0	1	1	0	3	3	1	1	0
**NG**		**1**	0	0	0	0	0	1	0	0	0
**MG**			**1**	0	0	0	1	0	0	0	0
**MGI**				**2**	1	0	1	1	0	2	0
**MH**					**19**	2	14	16	3	3	5
**UU**						**23**	2	13	2	0	3
**UP**							**175**	103	18	3	17
**GV**								**184**	22	4	16
**CA**									**41**	1	2
**TV**										**10**	1
**HPV**											**34**
**B**											
**Frequency of pathogens coinfections**											
	**CT**	**NG**	**MG**	**MGI**	**MH**	**UU**	**UP**	**GV**	**CA**	**TV**	**HPV**
Monoinfection	0	0	0	0	0	7	46	48	7	3	6
Coinfection	4	1	1	2	19	16	129	136	34	7	28

Table 1A presents the total number of each pathogen and the concomitant presence of different pathogens. The number in bold (each column read vertically) indicates the total number of women positive for the each pathogen. Coinfection of pathogens were commonly observed (each raw read horizontally).

Table 1B, (each column read vertically), shows the total number of each pathogen when detected as a monoinfection and coinfection.

*CT C.trachomatis, NG N.gonorrhoeae*, *MG M.genitalium, MGI M.girerdii, MH M.hominis, UU U.urealyticum, UP U.parvum, GV G.vaginalis*, *CA C.albicans, TV T.vaginalis*

Urogenital pathogens were isolated from women as either single infection or coinfections. The presence of a single pathogen was recovered from 140 women (28%)*.* The remaining urogenital pathogens were diversely recovered from women as two (34%, *N* = 129), three (6.5%, *N* = 33), four (1.8%, *N* = 9) or six coinfections (0.2%, *N* = 1) (Table 1).

### Association between pathogens and symptoms

#### In symptomatic patients

Among the 505 women, 50% (*N* = 257) reported having genital clinical symptoms, of whom 64% (*N* = 165) were positive for the tested pathogens. A high rate of *U.parvum* and *G.vaginalis* coinfection was observed (*N* = 103), among which only 49% were seen as symptomatic. No significant association was however observed between the presence of studied pathogens (when detected as single or multiple infections) and the presence of any symptoms (*p* = 0.330).

#### In asymptomatic patients

**Fig. 1:**
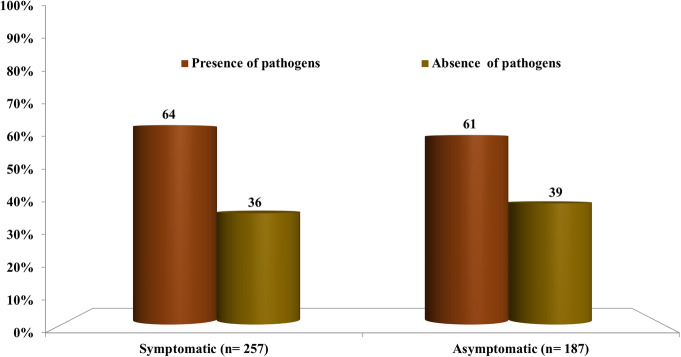
Percentage of pathogens isolated from symptomatic and asymptomatic women

Lack of symptoms was reported in 37% (*N* = 187) of the women, of whom 61% (*N* = 114) were carrying the pathogens (Fig. 1). The most commonly isolated pathogens from these 114 patients were *U. parvum, G.vaginalis*, *C. albicans and U. urealyticum* with 54, 37, 33 and 29%, respectively.

### Rates of HPV genotypes

**Fig. 2:**
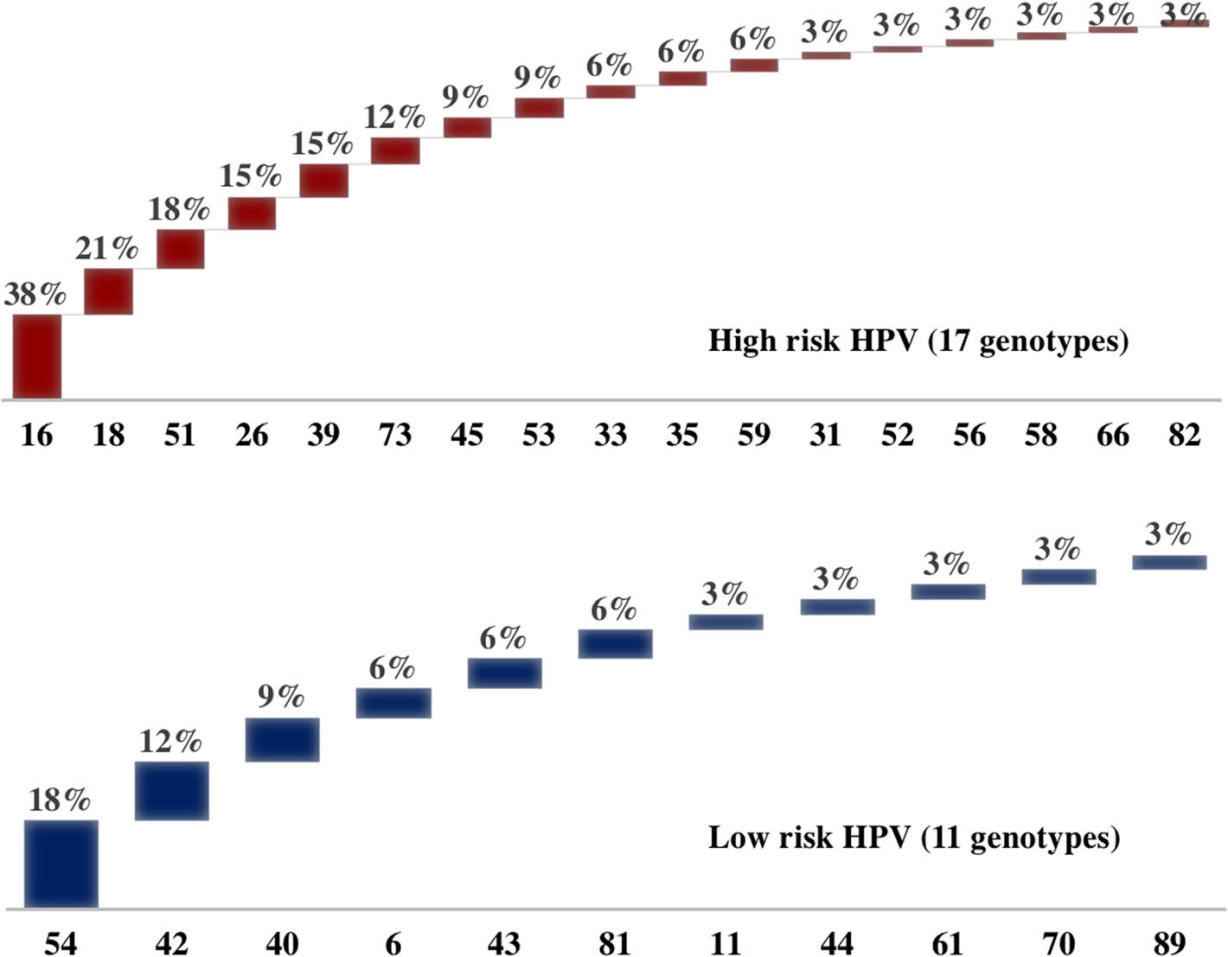
Distribution of High Risk and Low Risk HPV isolated from 34 women

The HPV positive samples (*N* = 34) had varied HPV genotype combinations of HR-HPV and LR-HPV **(**Table S2**)** with different prevalence rates and percentages. Figure 2 reveals the presence of 17 HR-HPV genotypes detected in the samples; the highest rates being recorded for types 16 (38%, *N* = 13) and 18 (21%, *N* = 7). A lower prevalence of LR-HPV was observed where 11 LR-HPV genotypes were identified, with the highest number of patients being recorded for types 54 (17.6%, *N* = 6) and 42 (11.7%, *N* = 4). HPV genotypes were grouped on the basis of their presence as single, double, triple or more genotypes (Table S2); the highest percentage was observed as a single HPV genotype reaching 44% (*N* = 15). HPV genotype 16 was found as a single genotype in five women (33%). As the number of coinfecting HPV genotypes increases, the percentage of women positive for more than one genotype decreases from 24% (*N* = 8) for 2 types, to 12% (*N* = 4) for 3 types, reaching 3% (*N* = 1) for 8 simultaneous genotypes (Table S2). The authors were not able to propose a pattern of HPV genotypes prevalence among positive samples that has reached a statistical significance (Table S2).

### Pap test and HPV

Further investigations were conducted for the 17 HPV positive patients who had PAP smear.

Their age ranged between 21 and 39 years old with the majority being 21 and 29 years (*N* = 12, 71%). Five patients had normal cytological results in the presence of HR-HPV (Table S3). The remaining patients had cytological changes in the presence of HPV.

### HPV vaccination in HPV infected women

From the 505 participants, 20 (4%) had taken HPV vaccine. Of the vaccinated women, 7 (35%) were positive for HPV. By observing the genotype combination present in the 7 women, two had one of the HR-HPV genotypes present in all HPV vaccines; one female was positive for HR-HPV type 16 and another for HR-HPV type 18 along with other HPV genotypes. Three women were infected with LR-HPV genotypes.

### Demographic characteristics of the sample and their association with STI’s

A total of 505 women aged between 18 and 69 years, with a mean age of 38 years (SD = 10.8), were enrolled in this study; a third of the women who participated were between the ages of 30 to 39 years old (32.8%). The majority were Lebanese (63.6%) and housewives (63.6%) (Table 2). As for the marital status, a high number of women were married (86.2%). Most of the women (77.8%) reported having had only one sexual partner while 0.01% had more than three.

**Table 2:** Demographic characteristics and behavioral risk factors of the 505 women in the sample

	Total (%) N	Positive for STIs (%) N
**Nationality (*N*** **= 455)**		
Lebanese	63.3% (288)	57.9% (167)
Syrian	24.2% (110)	67.2% (74)
Palestinian	8.8% (40)	60% (24)
Other	3.7% (17)	82.3% (14)
Missing	50	
**Age Range (*N*** **= 448)**		
18–29	27.5% (123)	73.9% (91)
30–39	32.8% (147)	59.1% (87)
40–49	23.4% (105)	62.8% (66)
50–69	16.3% (73)	43.8% (32)
Missing	57	
**Employment (*N*** **= 431)**		
None (housewife)	63.6% (274)	59.4% (163)
Yes	6.0% (26)	66.9% (17)
Missing	74	
**Marital Status (*N*** **= 486)**		
Single	7.6% (37)	86.4% (32)
Divorced/Widowed	6.2% (30)	40% (12)
Currently Married	86.2% (419)	60.6% (254)
Missing	19	
**Behavioral Risk Factors**		
**Alcohol (*N*** **= 487)**		
Yes	16.2% (79)	73.4% (58)
No	83.7% (408)	58.8% (240)
Missing	18	
**Smoking (*N*** **= 488)**		
Yes	32.1% (157)	66.2% (104)
No	67.8% (331)	58.9% (195)
Missing	17	
**Contraceptive Use (*N*** **= 458)**		
Yes	36.5% (167)	65.2% (109)
No	63.5% (291)	58.4% (170)
Missing	47	
**Contraceptive Type (*N*** **= 162)**		
Intrauterine Device	53.7% (87)	71.2% (62)
Pills	27.2% (44)	63.% (28)
Male Condom	17.9% (29)	44.8% (13)
Loop	0.6% (1)	100% (1)
Female Condom	0.6% (1)	0% (0)
Missing	5	

### Sociodemographic and behavioral risk factors and their association with STIs

There was a statistically significant association between marital status (p = 0.000) and the presence of infection. Eighty-six percent (86%) of single women had an infection compared to 61% of married women and 40% of divorced/widowed women. No association was observed between nationality and the presence of infection.

A significant association was observed between the presence of infection and age groups, with a higher proportion among younger women aged between 18 and 29 (73.9%) compared to older women (50 to 69) (43.8%) (*p* = 0.000).

**Fig. 3:**
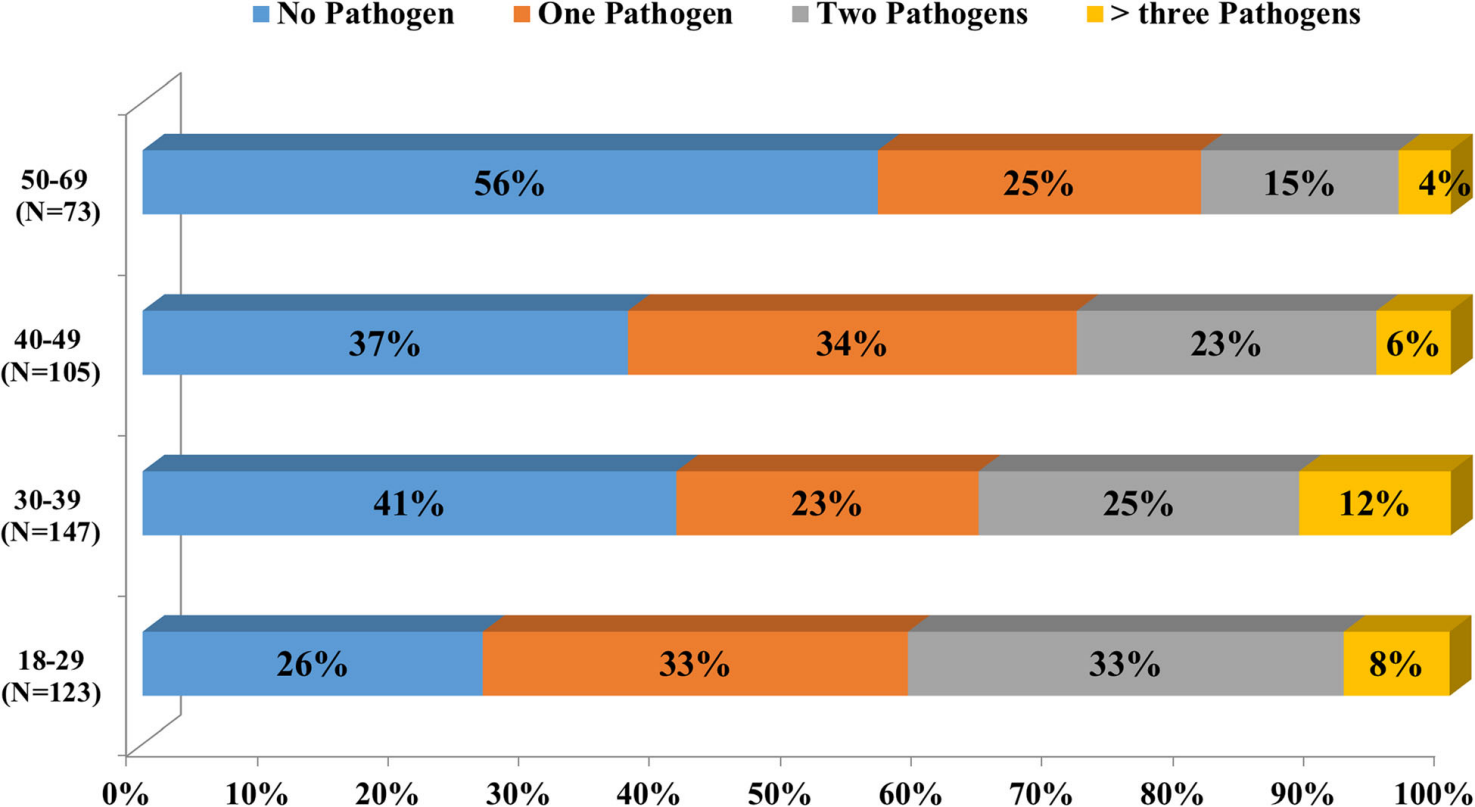
Percentage of single or multiple pathogens isolated from women in different age groups

We have seen that *C.albicans* was prominent in the age group 30–39 (*p* = 0.058). Coinfections were mostly observed in women aged between 18 and 29 years, while women aged 50 and above were either not colonized (56%) by the studied pathogens or colonized by a single pathogen (25%) (Fig. 3). However, no association was observed between the age group and coinfections.

Among the 34 women infected with HPV, 14 ranged between 21 and 29 years (median age of 30 years). Moreover, it was noticed that as age increases the presence of HPV decreases: 11 women ranged between 30 and 39 years, 5 women between 40 and 49 years and 4 women between 50 and 59 years.

Other findings revealed that a combination of ≥3 HPV genotypes were most likely found in age groups 20–29 and 30–39 while older age groups, 40–49 and 50–59, had either one or two genotypes (Table S2).

Women who reported to smoke and drink alcohol were 32 and 16% respectively. The only significant association between behavioral risk factors and the presence of infection was the consumption of alcohol with 73% being infected among consumers compared to 59% among non-consumers (*p* = 0.015).

Only 162 women (32%) were using contraceptives, with intrauterine device being the most frequently used (*N* = 87, 53.7%). The presence of infection was significantly associated with the type of contraceptive (*P* = 0.036), with a higher proportion among IUD users.

Table 3 presents univariate and multivariate logistic regression results for the presence of infection with the demographic and behavioral characteristics which appeared to be significant at the bivariate level. The univariate logistic regression odds ratio showed that being older or ever married were protective, while consuming alcohol or using an IUD were risk factors. In the multivariate analysis, after controlling for all characteristics significant at the bivariate level, only younger age and the use of IUD remain risk factors for infection. In fact, women appear to be 3 times more at risk of infection if they are between the ages of 18–29 compared to women older than 50. The use of an IUD more than doubles the risk of infection for women.

**Table 3:** Adjusted and unadjusted logistic regression results for the association between presence of infection and select demographic and behavioral risk factors (*n* = 373)

Variable	Unadjusted Results			Adjusted Results^**1**^		
	OR	CI	p	OR	CI	p
**Age**						
18–29	3.644	1.973–6.727	0.000	3.005	1.486–6.073	**0.002**
30–39	1.858	1.053–3.277	0.032	1.553	0.813–2.967	0.182
40–49	2.168	1.180–3.985	0.013	1.806	0.915–3.565	0.089
50–69	1.000					
**Marital status**						
Single	1.000					
Married	0.241	0.092–0.630	0.004	0.364	0.087–1.526	0.167
Divorced/widowed	0.104	0.032–0.343	0.000	0.211	0.041–1.081	0.062
**Alcohol consumption**	1.933	1.130–3.307	0.016	1.309	0.632–2.709	0.468
**IUD use**	1.781	1.071–2.963	0.026	2.024	1.158–3.537	**0.012**

Note: *OR* odds ratio; *95%CI* 95% confidence interval

1 model includes age, marital status, alcohol consumption and IUD use.

## Discussion

The aim of the present epidemiological study was to investigate the prevalence of STIs among women using a highly reliable, sensitive and cost effect diagnostic test for simultaneous detection of microorganisms. The results showed that STI pathogens are found in both symptomatic and asymptomatic women. Clinical management and treatment of these pathogens in asymptomatic individuals are challenging. On the other hand, the absence of pathogens in the presence of symptoms could be explained by the presence of microbiomes not yet identified or by disease of non-microbial origin.

### Low prevalence of C. trachomatis and N. gonorrhea

This study presented a low prevalence of both *C.trachomatis* (0.8%) and *N.gonorrhoeae* (0.2%) including one asymptomatic coinfection. Our results were comparable to other studies in the Middle East and North Africa region (MENA) and the Eastern Mediterranean Region (EMR) which reported a prevalence ranging from 2.5 to 4.2% for *C.trachomatis* and 0.1 to 0.6% for *N.gonorrhoeae* [18–22]. The prevalence of both bacteria are reported to be higher in UK, France and American regions [19, 20, 23–27]. The low prevalence of these infections in Lebanon may relate to the fact that the majority of women were married and probably faithful to their husbands; few women reported to have multiple sexual partners.

### Prevalence of other *genital pathogens*

Our study has found that among the mollicutes, *U.parvum* was the most frequently detected microorganism with an overall prevalence of 34.9%, and the most isolated single pathogen. *U.parvum* was frequently associated with increased positivity for *G.vaginalis*, however, no significant association was found between the detection of both pathogens and symptoms. Neither *U.parvum, U.urealyticum*, nor *G.vaginalis* seem to be significantly associated with symptoms in females when isolated as a single pathogen; their role in the female urogenital tract therefore remains unknown, although reported cases have shown that these infections can cause cervicitis and bacterial vaginosis [28–30].

*C.albicans*, whose overall prevalence was lower than *G.vaginalis*, was not associated with symptoms and was mostly observed among women aged between 30 and 39 years (50%). *Candida* species may persist for months, years and live in symbiosis with vaginal microbiota without causing any symptoms [31, 32]. However, acute vulvovaginal candidiasis can occur following colonization when there is breakdown in the symbiosis, which triggers the overgrowth of C*andida* or alteration in the protective defense mechanisms [33, 34].

New diagnostic assays such as nucleic acid testing improved the detection of *T.vaginalis* especially in asymptomatic individuals [35]. This may explain why the prevalence of *T.vaginalis* infection in our study was higher (2%) than the one found in a previous study conducted in Lebanon (1.2%) [36]. *T. vaginalis* was also reported to be low in other industrialized countries [21, 37].

### Genotypic distribution of HPV genotypes

HPV genotypes were investigated to provide an insight into the need for HPV vaccination at sexual debut or early adolescence age. The prevalence of HPV in our study sample was 6.7% which is in concordance with the results of a meta-analysis that was conducted in 2012 on HPV in the MENA region [38]. The age distribution of HPV infection in the current study reflects the natural history of HPV infection. HPV rates peaked at the age of 20 to 29 (47%) almost after the start of the women sexual activity [39]. However, the HPV infection in women of a young age is often temporary and is cleared by their immune system. The occurrence of HPV was reported in 10% of women aged between 50 and 69. The latter could be explained by new acquisitions of the virus following a new sexual partner, changes in sexual practices, reactivation of the latent uncleared infection due to reduced immunity with aging or survival bias due to death of women who developed cervical cancer [40].

Data on the prevalence and genotype distribution of HPV infection in Lebanon are limited. The most frequently detected HPV genotypes among women were the HR-HPVs. Our study found that HPV16 (38%), HPV 18 (21%) HPV51 (18%), HPV 26 (15%), HPV39 (15%), and HPV 73 (12%) were the six predominant genotypes in the sample. If the immune system fails to clear the HR-HPV types, infections will be associated with high risk for cervical disease progression leading to cervical cancer [41].

WHO has issued a document on controlling cancer in the Eastern Mediterranean region where only 35% of cervical cancer were reported at early stages while the rest were discovered at later stages; the latter are usually associated with bad prognosis and minimal recovery rate [42]. Another meta-analysis study, conducted at the international level, showed that at an unspecified point in time, 11.7% of women with normal cervical cytological findings were positive for HPV infection; of which 70% were carcinogenic HPV types. The main variables affecting such data were age and geography [39]. In our study, five women had normal cytology in the presence of HR-HPV. In Lebanon, most clinicians use cytology screening for cervical cancer once a year instead of HR HPV-based screening, which may affect the early detection and treatment of women. Adding to that, the national prevalence of “ever used” of the PAP smear is 35% [43]. Cotesting PAP test and HR-HPV in women aged 30 years and older is more sensitive in detecting abnormal cells or cervical cancer than a Pap test alone; both are essential to decrease cervical cancer incidence in the Mediterranean region [44, 45]. Awareness campaigns on the advantages of early detection using contesting, particularly among vulnerable women, would reduce the incidence of cervical cancer.. Health care providers should insight women to regularly perform their screening test.

### Prevalence of HR-HPV genotypes and efficacy of HPV vaccine

In any country, it is necessary to know the HPV genotype distribution to assess the effectiveness of vaccines recommended by the gynecologists. The predominance of HR-HPV in our study favors the recommendation of HPV vaccination. Currently available HPV vaccines are Gardasil, Gardasil 9, and Cervarix [46, 47]; all 3 vaccines prevent infections with HPV types 16 and 18. Although Gardasil 9 protects against infection with additional HPV types such as 6, 11, 31, 33, 45, 52 and 58; it excludes other types of infections prevalent in the region such as type 51, 26, 39 and 73. According to the International Agency for Research on Cancer, HPV 51 and HPV 26 has been defined as human carcinogens [48]. Cross protection of Gardasil 9 against these various types should be further investigated.

HPV vaccines are still expensive in Lebanon, not yet recommended by all pediatricians and not offered by the Lebanese government as part of the national immunization program. The ministry of health in Lebanon should implement a systemic approach for HPV vaccination similar to that initiated by an international organization, GAVI, who helped 30 countries to introduce HPV vaccines into their national immunization programs with the aim to protect around 40 million girls from cervical cancer by 2020 [49, 50].

### Association between risk factors and infections

In this study, STIs were common in single (86%) and married women (61%) indicating that sexual behavior could be associated with infection [51]. Women may possibly engage in risky sexual acts to achieve motherhood or to sexually satisfy their partner. More information about women’s sexual behavior was hindered by the taboo around sexual disclosure. In the current study, the total number of single women who participated was low since the annual gynecologic exam as a routine checkup for single women is not a common practice in Lebanon.

The use of IUD in women was high reaching 54%; 72% of those were positive for the infection. Previous studies showed an association between IUD and pelvic inflammatory diseases among women with sexually transmitted infection [52]. The risk may vary depending on when the IUD was inserted, whether prior to bacterial exposure or afterwards. The IUD can be safe and effective if inserted under aseptic conditions.

This study has also shown an association between STIs and alcohol consumptions (73%). The latter could have an impact on the sexual behavior of women and their sexual arousal [51, 53–56].

### Strengths and limitations

In Lebanon and the MENA region, this is the first study that presents data about the prevalence of STIs among women by simultaneous detection of 11 pathogens and describes the distribution of circulating HR-HPV and LR-HPV genotypes. It also identifies various risk factors associated with STIs.

However, the prevalence of other STIs such as HSV, *Hemophilus ducreyi*, *Treponema pallidum* and blood born viruses (HBV, HCV and HIV) were not investigated. The sample chosen is convenient and limited in its majority to married women, it also excluded non pregnant women. Future research directions should include STI screening for men and women of different age groups at the national level.

## Conclusion

The results of this study should trigger the healthcare professionals to implement reproductive healthcare services that are accessible and affordable for all. Awareness campaigns are also necessary for the population to adopt a healthy sexual behavior.

## Supplementary information

**Additional file 1: Table S1.** Nucleotide sequences of primers and probes

**Additional file 2: Table S2.** HPV genotypes distribution patterns detected in 34 samples

**Additional file 3: Table S3.** Comparison of Pap smear results with HPV genotyping findings of 17 HPV positive patients

## Abbreviations

STIs: Sexually transmitted infections (STIs)
CDC: Centers for Disease Control
WHO: World Health Organization
MENA: Middle East and North Africa
HPV: Human papilloma virus
CT: Chlamydia.trachomatis
NG: Neisseria.gonorrhoeae
MG: Mycoplasma.genitalium
MGI: Mycoplasma.girerdii
MH: Mycoplasma hominis
UU: Ureaplasma.urealyticum
UP: Ureaplasma parvum
GV: Gardnerella vaginalis
CA: *Candida albicans*
TV: Trichomonas vaginalis
PAP: Papanicolaou
HR-HPV: High risk-human papilloma virus
LR-HPV: Low risk- human papilloma virus
PCR: Polymerase Chain reaction
IUD: Intrauterine device
OR: Odds ratio
CI: Confidence interval
HBV: Hepatitis B virus
HCV: Hepatitis C virus
HIV: Human immunodeficiency virus
GAVI: Global Alliance for Vaccines and Immunisation
