# Supplementary material for: Molecular epidemiology and socio-demographic risk factors of sexually transmitted infections among women in Lebanon
Source: BMC Infect Dis. 2020 May 27;20:375. doi: 10.1186/s12879-020-05066-8 (PMC7251815; doi:10.1186/s12879-020-05066-8)
Supplement: Supplementary file 1 — Additional file 1: Table S1. Nucleotide sequences of primers and probes [file 12879_2020_5066_MOESM1_ESM.docx]

| **Type of assay  (Target genes)** | **Primer/Probe** | **Sequences** | **References** |
| --- | --- | --- | --- |
| **Quadriplex assay**   MG (Adhesin P1, MgPa) UU (Urease) UP (Urease) TV (Repeated sequence) | MG FW | 5’- GAGAARTACCTTGATGGTCAGCAA-3’ | [[15](#_ENREF_15)] |
|  | MG RV | 5’- GTTAATATCATATAAAGCTCTACCGTTGTTATC-3’ |  |
|  | **MG-P** | **5’-FAM-ACTTTGCAATCAGAAGGT- MGB-3’** |  |
|  | UP-UU FW | 5’- AAGGTCAAGGTATGGAAGATCCAA-3’ |  |
|  | UP-UU-RV | 5’-TTCCTGTTGCCCCTCAGTCT-3’ |  |
|  | **UP-P** | **5’- CY5-TCCACAAGCTCCAGCAATTTG- BHQ2-3’** |  |
|  | **UU-P** | **5’-HEX-ACCACAAGCACCTGCTACGATTTGTTC-BHQ1-3’** |  |
|  | TV FW | 5’- CATTGACCACACGGACAAAAAG-3’ |  |
|  | TV RV | 5’- CGAAGTGCTCGAATGCGA-3’ |  |
|  | **TV-P** | **5’-Rox-TCATTTCGGATGGTCAAGCAGCCA-BHQ2-3’** |  |
| **Quadriplex assay**   CT (Plasmid/ MOMP) NG (PorA) IC (Synthetic DNA) | CT P FW | 5´-AACCAAGGTCGATGTGATAG-3´ | [[15](#_ENREF_15)] |
|  | CT P RV | 5´-TCAGATAATTGGCGATTCTT-3´ |  |
|  | **CT- P-P** | **5´-FAM-CGAACTCATCGGCG-MGB-3'** |  |
|  | CT-M FW | 5´-GACTTTGTTTTCGACCGTGTT-3´ |  |
|  | CT-M RV | 5´-ACARAATACATCAAARCGATCCCA-3´ |  |
|  | **CT-M-P** | **5´-HEX-ATGTTTACVAAYGCYGCTT-MGB-3´** |  |
|  | NG FW | 5´-CCGGAACTGGTTTCATCTGATT-3' |  |
|  | NG RV | 5´-GTTTCAGCGGCAGCATTCA-3´ |  |
|  | **NG-P** | **5´-ROX-CGTGAAAGTAGCAGGCGTATAGGCGGACTT-BHQ2- 3´** |  |
|  | IC FW | 5′-GTGCTCACAC CAGTTGCCGC-3 |  |
|  | IC RV | 5′-GCTTGGCAGC TCGCATCTCG-3′ |  |
|  | **IC-P** | **5′-CY5-ATTGTGTGGGTGTGGTGTGGGTGTGTGC- BHQ3-3′** |  |
| **Duplex Assay**  MH (16S rRNA) MGI (16S rRNA) | MhF FW | 5’-TTTGGTCAAGTCCTGCAACGA-3’ | [[16](#_ENREF_16)] |
|  | MhR RV | 5’-CCCCACCTTCCTCCCAGTTA-3’ |  |
|  | **MH-P** | **5’-HEX-TACTAACATTAAGTTGAGGACTCTA-BHQ1-3’** |  |
|  | MGi-FW | 5’-GCAAGTCGAGCGGAGGTAGC-3’ |  |
|  | MGi-FW | 5’-ACTTTCATGCGAATGTATATCATATAAGG-3’ |  |
|  | **MGi-P** | **5’-CY5-TTCGCCGCTAAGTGTATTG-BHQ2-3’** |  |
| **Duplex Assay**  GV (Tuf) CA (RNAse P) | GV FW | 5'- TCCCAACCCCAACTCACGATCTT-3' | [[17](#_ENREF_17), [18](#_ENREF_18)] |
|  | GV RV | 5'-CGCAAACCAACRATCTCAACTGG-3' |  |
|  | **GV-P** | **5'-CY5-CCATCTCCGGTCGTGGTAC-BHQ1-3'** |  |
|  | CA FW | 5'- CGGGTGGGAAATTCGGT-3' |  |
|  | CA RV | 5'-CAATGATCGGTATCGGGT-3' |  |
|  | **CA-P** | **5'-FAM-CAGCTTGTAGTAAAGAATTACTCAC-BHQ1-3'** |  |

CT*:Chlamydia trachomatis*, NG:*Neisseria gonorrhoeae,* MG:*Mycoplasma genitalium*, UU:*Ureaplasma urealyticum*, UP:*Urealplasma parvum*, TV:*Trichomonas vaginalis*, MH:*Mycoplasma hominis*, MGI:*Mycoplasma girerdii*, GV:*Gardnerella vaginalis,* CA:*Candida albicans*.

Table S1: Nucleotide sequences of primers and probes
