# Supplementary material for: Molecular epidemiology and socio-demographic risk factors of sexually transmitted infections among women in Lebanon
Source: BMC Infect Dis. 2020 May 27;20:375. doi: 10.1186/s12879-020-05066-8 (PMC7251815; doi:10.1186/s12879-020-05066-8)
Supplement: Supplementary file 2 — Additional file 2: Table S2. HPV genotypes distribution patterns detected in 34 samples [file 12879_2020_5066_MOESM2_ESM.docx]

|  | **High risk** | **Low risk** | **Total number  of samples** |
| --- | --- | --- | --- |
| **Single HPV genotype**  **(N=15)** | 16 |  | 5 |
|  | 18 |  | 1 |
|  | 26 |  | 1 |
|  | 33 |  | 1 |
|  | 39 |  | 1 |
|  | 45 |  | 1 |
|  |  | 11 | 1 |
|  |  | 61 | 1 |
|  |  | 70 | 1 |
|  |  | 81 | 1 |
|  |  | 89 | 1 |
| **Two HPV genotypes**  **(n=8)** | 16 | 54 | 1 |
|  | 16 | 42 | 1 |
|  | 16, 26 | - | 1 |
|  | 16, 52 | - | 1 |
|  | 18, 35 | - | 1 |
|  | 18, 51 | - | 1 |
|  | 31 | 6 | 1 |
|  | 53 | 54 | 1 |
| **Three HPV genotypes**  **(n= 4)** | 16, 58 | 54 | 1 |
|  | 16, 73 | 54 | 1 |
|  | 16, 35, 59 | - | 1 |
|  | 39, 53, 73 | - | 1 |
| **> Three HPV genotypes**  **(n= 7)** | 18, 51, 56 | 54 | 1 |
|  | 18, 26, 39 | 40 | 1 |
|  | 16, 26, 45, 51 | 81 | 1 |
|  | 16, 59, 73, 82 | 6, 44 | 1 |
|  | 18, 26, 39, 73 | 40, 42 | 1 |
|  | 51, 53, 66 | 40, 42, 43 | 1 |
|  | 18, 33, 39, 45, 51 | 42, 43, 54 | 1 |

**Table S2: HPV genotypes distribution patterns detected in 34 samples**
