# Supplementary material for: Molecular epidemiology and socio-demographic risk factors of sexually transmitted infections among women in Lebanon
Source: BMC Infect Dis. 2020 May 27;20:375. doi: 10.1186/s12879-020-05066-8 (PMC7251815; doi:10.1186/s12879-020-05066-8)
Supplement: Supplementary file 3 — Additional file 3: Table S3. Comparison of Pap smear results with HPV genotyping findings of 17 HPV positive patients [file 12879_2020_5066_MOESM3_ESM.docx]

| **Sample #** | **High-risk HPV** | **Low-risk HPV** | **Inflammation**  **status** | **ASCUS/HSIL/LSIL** | **Other clinical findings** |
| --- | --- | --- | --- | --- | --- |
| 1 | 18, 51, 56 | 54 | Positive |  | Squamous metaplasia |
|  | 16 | 54 |  |  | Normal Cytology |
| 2 | 51, 53, 66 | 40, 42, 43 | Positive | ASCUS | Epithelial Cell Abnormality (ECA) |
| 3 | 16 | - | Positive |  | Parakeratosis |
| 4 | - | 61 | Positive |  | Normal Cytology |
| 5 | 18, 26, 39 , 73 | 40, 42 | Positive |  | Parakeratosis & Squamous Metaplasia |
| 6 | 16, 26, 45, 51 | 81 |  | ASCUS | Epithelial Cell Abnormality |
| 7 | 16, 73 | 54 | Positive |  | Parakeratosis |
| 8 | 39, 53, 73 | - |  | HSIL | ECA & Moderate Dysplasia |
| 9 | 33 | - |  |  | Normal Cytology |
| 10 | 16, 59, 73, 82 | 6, 44 | Positive |  | Normal Cytology |
| 11 | 16 | - |  | LSIL | Epithelial Cell Abnormality |
| 12 | - | 89 | Positive |  | Normal Cytology |
| 13 | 18, 33, 39, 45, 51 | 42, 43, 54 | Positive |  | Parakeratosis & Benign Cellular changes |
| 14 | 53 | 54 | Positive |  | Normal Cytology |
| 15 | - | 81 | Positive |  | Squamous metaplasia |
| 16 | 16, 58 | 54 | Positive | LSIL (CIN I-II) | Epithelial Cell Abnormality |
| 17 | 16, 35, 59 | - | Positive |  | Normal Cytology |

ASCUS: Atypical squamous cells of undetermined significance; LSIL: Low-grade squamous intraepithelial lesion; HSIL: High-grade squamous intraepithelial lesion is a higher level than LSIL; CIN: Cervical intraepithelial neoplasia

**Table S3: Comparison of Pap smear results with HPV genotyping findings of 17 HPV positive patients**
